# Zearalenone Depresses Lactation Capacity Through the ROS-Mediated PI3K/AKT Pathway

**DOI:** 10.3390/ani15071050

**Published:** 2025-04-04

**Authors:** Hong Chen, Di Qiu, Xue Miao, Wenyue Yang, Qi He, Hao Ren, Luyao Zhang, Hongri Ruan, Jiantao Zhang, Na Zhang

**Affiliations:** 1 College of Veterinary Medicine, Northeast Agricultural University, Harbin 150030, China; dnchenhong909@163.com (H.C.); qd13836005047@163.com (D.Q.); miaoxue1997@163.com (X.M.); 15546348598@163.com (W.Y.); 18846770949@163.com (Q.H.); a985743763@163.com (H.R.); zhangluuuyao@163.com (L.Z.); 15776581316@163.com (H.R.); 2 Heilongjiang Provincial Key Laboratory of Pathogenic Mechanism for Animal Disease and Comparative Medicine, Harbin 150030, China; 3 Key Laboratory of Dairy Science, Ministry of Education, Northeast Agricultural University, Harbin 150030, China

**Keywords:** mammary gland, milk production, oxidative stress, proliferation, zearalenone

## Abstract

The effects of zearalenone (ZEA), a fungal toxin in food and feed, remain unclear on the mammary gland and lactation. This study examines ZEA-induced damage in lactating mice and bovine mammary epithelial cells (MAC-T), focusing on the role of the phosphatidylinositol 3-kinase/protein kinase B (PI3K/AKT) pathway in regulating cell proliferation and apoptosis. The results demonstrated that exposure to ZEA at different doses (5 mg/kg, 10 mg/kg, and 20 mg/kg) reduced lactation in female mice and slowed weight gain in their offspring. Hematoxylin and eosin (HE) staining and CSNK immunofluorescence staining of mammary tissue confirmed ZEA-induced mammary gland damage in vivo. Further analysis using PCNA immunohistochemistry and fluorescent TUNEL staining revealed that ZEA promoted apoptosis and decreased the proliferative capacity of mammary tissues. In vitro, 20 μM ZEA decreased MAC-T cell proliferation, increased apoptosis and oxidative stress, inhibited PI3K/AKT signaling, and decreased κ-casein (CSNK) expression. Pretreatment with a reactive oxygen species (ROS) scavenger (NAC) or PI3K/AKT activator (740-Y-P) reversed these effects, with NAC specifically restoring PI3K/AKT activity inhibited by ZEA. Overall, this study concludes that ZEA induces MAC-T cell apoptosis and disrupts proliferation via the ROS-mediated PI3K/AKT pathway, ultimately impairing lactation function. These findings highlight potential targets for managing ZEA contamination in food and its impact on lactation.

## Simple Summary

Zearalenone, a prevalent mycotoxin in food and feed, accumulates in the liver, kidneys, and animal products, posing significant health risks to humans and animals. However, its effects on mammary gland damage and lactation capacity remain unclear. This study investigated zearalenone’s impact on lactating mice and bovine mammary epithelial cells, focusing on the role of the PI3K/AKT pathway in regulating cell proliferation and apoptosis. In vivo experiments revealed that zearalenone reduced lactation in female mice, slowed offspring weight gain, increased mammary tissue apoptosis, and decreased proliferation. In vitro, 20 μM zearalenone inhibited cell proliferation, increased apoptosis and oxidative stress, suppressed PI3K/AKT signaling, and reduced κ-casein expression. These effects were reversed by pretreatment with reactive oxygen species scavengers or PI3K/AKT activators. Overall, ZEA induces apoptosis and disrupts proliferation through the ROS-mediated PI3K/AKT pathway, ultimately impairing lactation. Controlling zearalenone exposure could enhance milk production in animals, benefiting the dairy industry and ensuring safer food supplies for consumers.

## 1. Introduction

It is estimated that 60% to 80% of crops worldwide are contaminated per year by mycotoxins, which are secondary metabolites produced by fungi when plants are growing, developing, harvesting, and processing grain [1]. These compounds are significant environmental pollutants that threaten food and feed safety. To date, about 400 natural mycotoxins have been identified [2], including aflatoxin, zearalenone (ZEA), fumonisin, deoxynivalenol, and T-2 toxin. ZEA, a non-steroidal estrogenic mycotoxin, is biosynthesized by *Fusarium graminearum* through the polyketide pathway [3]. ZEA contamination commonly affects corn, soybean meal, and wheat, which are widely used in animal feed globally [4]. Toxicokinetic studies indicate that ZEA is rapidly and efficiently consumed by the gastrointestinal tract in humans and many domestic animals [5]. ZEA has been found to persist in dairy cows for at least 28 days when they are fed ZEA-contaminated feed at 0.05 mg/kg, with residues detectable in milk [6]. ZEA has also been shown to accumulate in various tissues, including the liver, kidneys, reproductive organs, and related products, such as eggs and meat [7,8]. Therefore, the widespread nature of ZEA, together with its high absorption and its long half-life, leads to its bioaccumulation via higher consumption than elimination in the organism, and may even biomagnify, thus posing a threat to human health [9,10,11,12]. Recently, studies have reported that ZEA has been detected in the serum, urine, and milk of humans, cows, and pigs [13,14,15]. Additionally, ZEA and its metabolites have been shown to cause DNA fragmentation, cell cycle arrest, and chromosomal aberrations in vivo, resulting in reproductive toxicity [16], immunotoxicity [17], genotoxicity [18], and carcinogenicity [19]. It was shown that ZEN exposure induced programmed cell death in lactogen-stimulated MAC-T cells [20]. However, it is unknown whether ZEA affects mammary gland function and milk production performance in lactating animals, as well as the specific mechanisms underlying mammary gland damage.

The phosphatidylinositol 3-kinase/protein kinase B (PI3K/AKT) signaling pathway plays a crucial role in maintaining cellular homeostasis and has been implicated in cell processes related to cell proliferation, differentiation, and apoptosis [21]. For instance, activation of the PI3K/AKT pathway regulates cell cycle proteins in mouse myoblasts, facilitating cell proliferation and differentiation [22]. Selenium, a key component of the antioxidant system, has been demonstrated to inhibit the PI3K/AKT pathway when dysregulated, leading to apoptosis in mouse testicular cells [23]. Additionally, the PI3K/AKT pathway is an established target of oxidative stress, with reactive oxygen species (ROS) inducing cell death by negatively regulating this signaling cascade [24]. Studies also indicate that the PI3K/AKT pathway may be involved in regulating the synthesis of κ-casein (CSNK), a milk protein that constitutes 80% of total milk protein [25,26]. Moreover, a close relationship has been observed between CSNK levels and milk production [27,28]. Notably, ZEA has been shown to induce ROS accumulation, causing metabolic disturbances and oxidative damage to the mammary glands of dairy cows [29,30,31,32]. This ROS overproduction has been negatively correlated with CSNK synthesis, further linking oxidative stress to reduced milk protein synthesis [33].

Despite the increasing number of studies examining oxidative stress and apoptosis in mammary cells induced by ZEA, its impact on mammary gland damage and milk production performance in lactating animals, as well as the specific underlying mechanisms, remain largely unknown. Based on preliminary evidence that the ROS-mediated PI3K/AKT pathway is inhibited by ZEA, we hypothesized that (1) ZEA is capable of inducing apoptosis in mammary epithelial cells and inhibiting cellular proliferative capacity, which disrupts lactation performance, and (2) drugs that modulate ROS/PI3K/AKT signaling could rescue ZEA-induced mammary cell injury. Therefore, we utilized lactating female KM mice as an animal model and orally administered different doses of ZEA to study its effects on litter weight, milk yield, and mammary cell proliferation and apoptosis levels. Additionally, bovine mammary epithelial cells (MAC-T) were employed as an in vitro model. These cells were pretreated with a ROS scavenger (N-acetylcysteine, NAC) or a PI3K/AKT activator (740-Y-P) to examine their effects on ZEA-induced mammary gland injury. The findings from this study will deepen our understanding of the molecular mechanisms underlying ZEA-induced mammary gland damage and provide a theoretical foundation for the development of safe feed products and protective agents to mitigate ZEA toxicity.

## 2. Materials and Methods

### 2.1. Chemicals and Reagents

ZEA and 740-Y-P were obtained from MedChemExpress (Monmouth Junction, NJ, USA), while NAC was sourced from Solarbio (Beijing, China). The reagents utilized in the cellular experiments were dissolved in dimethyl sulfoxide (Solarbio, China), whereas the ZEA administered in the animal experiments was dissolved in olive oil. All reagents were stored at −20 °C.

### 2.2. Mouse Maintenance and Zearalenone Treatment

Animal experiments were approved by the Animal Ethics Committee of Northeast Agricultural University (Approval Number: NEAUEC202403128). KM mice (6 weeks old) were sourced from Liaoning Changsheng Biotechnology Co., Ltd. (Liaoning, China) and housed under controlled conditions of 25 ± 1 °C and 55 ± 5% relative humidity, with a 14 h light/10 h dark cycle, and provided ad libitum access to food and water. Following a one-week acclimatization period, male and female mice were housed together in 2:1 ratio, while pregnant females were housed individually until giving birth. One day post-delivery, litters were reduced to ten pups, and any female mice with less than ten pups were excluded. Ultimately, 20 female mice with similar delivery times (deviation of no more than 3 days) were selected for inclusion. These mice were randomly assigned to one of four groups (*n* = 5): (1) control group (Group C); (2) 5 mg/kg ZEA-exposed group (Group ZEA-L); (3) 10 mg/kg ZEA-exposed group (Group ZEA-M); and (4) 20 mg/kg ZEA-exposed group (Group ZEA-H). Mice in the ZEA-L, ZEA-M, and ZEA-H groups received different concentrations of ZEA via oral force-feeding daily after delivery, while those in Group C were administered equivalent amounts of olive oil for 14 days. The ZEA doses used in this study have been previously reported [34,35,36,37]. No mortality was observed during the trial. At mid-lactation, the mammary glands of the mice were fully developed, and the offspring were unable to consume the feed and water provided to their mothers. The total body weight of pups (*n* = 10) from the same litter was weighed daily. Additionally, on days 7 and 14 of lactation, milk production was measured in all female mice following published methods [38]. Briefly, all offspring were separated from their dams for 5 h. After this fasting period, the offspring were returned for a 90-min lactation session, during which no food or water was provided. The body weight of the entire litter was measured before and after lactation to assess changes.

### 2.3. Sample Collection and Histomorphological Observation

All female mice and their offspring were euthanized on day 14 post-lactation. The mammary tissues from female mice were fixed in 4% paraformaldehyde for 24 h before being embedded in paraffin. Next, they were sectioned and stained with H&E at a thickness of 5 μm. The prepared slides were subsequently scanned using a panoramic scanner (3DHISTECH LTD., Budapest, Hungary). Ten slides were selected from left to right at a magnification of 200× to observe histological changes in the mammary gland.

### 2.4. Immunohistochemistry

The mammary gland sections were dewaxed in xylene and rehydrated by decreasing the volume of ethanol. After cooling, citrate antigen retrieval solution was applied. The sections were incubated in 3% hydrogen peroxide solution for 25 min. Following this, 3% bovine serum albumin drops were added to the sections to block them, and they were incubated for 30 min. The slides were then incubated overnight at 4 °C with the antibody against proliferating cell nuclear antigen (PCNA) (1:5000, ABclonal, Shanghai, China). Afterward, the sections were treated at 37 °C for 45 min with secondary antibody (1:200, Servicebio, Wuhan, China), and stained with DAB for 1 to 5 min. Next, hematoxylin was added to the sections for 3 min, and they were re-blued with a hematoxylin re-blue solution. Ten slides were selected from each set, from left to right, at a magnification of 200×. Positively stained cells were counted by an expert using a blinded method. The integrated optical density value of positive staining was evaluated using ImageJ software (1.25a, 1.8.0 112, National Institutes of Health, Bethesda, MD, USA).

### 2.5. Tissue Immunofluorescence Staining

First, paraffin sections underwent dewaxing, rehydration, antigen retrieval, sealing, and primary antibody incubation as described in Section 2.4. Following overnight incubation with the primary antibody, the sections were treated with Alexa Fluor 488-labeled goat anti-rabbit IgG (1:400, Servicebio, China) for 45 min at 37 °C. Subsequently, they were incubated with DAPI staining solution for 10 min, and protected from light. Afterward, the slides were scanned using a panoramic scanner.

### 2.6. TUNEL Assay

Mammary gland sections were deparaffinized in xylene, then rehydrated in ethanol, followed by treatment with Proteinase K Repair Solution, then incubated with biotin-dUTP labeling mixture along with terminal deoxynucleotidyl transferase enzyme, protected from light. Following this, DAPI staining solution was applied, and the sections were incubated for an additional 10 min away from light. Ten slides were selected from each set, from left to right, at a magnification of 200×. Positively stained cells were counted by an expert using a blinded method.

### 2.7. Cell Culture

The MAC-T cells were generously provided by Heilongjiang Bayi Agricultural and Reclamation University. MAC-T cells were cultured in vitro in DMEM/F12 medium (Gibco, Carlsbad, CA, USA), supplemented with 10% fetal bovine serum (Biological Industries, Beit HaEmek, Israel) and 1% penicillin-streptomycin, and maintained in a cell culture incubator (Thermo, Waltham, MA, USA) at 37 °C with 5% CO_2_. For cell identification, MAC-T cells were immunofluorescently stained with cytokeratin 18, a characteristic marker of mammary epithelial cells, following previously established methods [39].

### 2.8. Cell Viability Assay

CCK-8 (APExBIO, Houston, TX, USA) was used for assessment of cell viability. Briefly, MAC-T cells were inoculated in 96-well plates at a density of 5000 cell number/well and cultured for 18–24 h. Then, the cells were exposed to ZEA (0, 1, 5, 10, 20, 30, 50, 100 μM) for 24 h. After the treatment, CCK-8 solution was added to each well, then incubated at 37 °C for 2 h. A microplate reader (BioTek, Burlington, VT, USA) was used to measure optical density (OD).

### 2.9. Lactate Dehydrogenase Assay

An LDH assay kit (Nanjing Jiancheng Bioengineering Institute, Nanjing, China) was used for evaluated cell membrane integrity. The groupings and administered doses were consistent with those described in Section 2.8. After exposure to ZEA for 24 h, cell culture medium was collected. Reagents and samples were added following kit instructions, and OD values were measured though the microplate reader.

### 2.10. Cell Proliferation Assay

Cell proliferation was evaluated using the BeyoClick™ EdU-555 kit (Beyotime, Shanghai, China), with 1.5 × 10^5^ cell number/well. After the administration treatment (5 mM NAC pretreatment for 1 h followed by ZEA incubation, 30 μM 740-Y-P incubated simultaneously with ZEA for 24 h), each well was treated by adding 2 × EdU working solution for 3 h. Cell proliferation was quantified by the number of BdU-positive cells/total cells observed using a fluorescence microscope (Leica, Wetzlar, Germany).

### 2.11. Detection of Oxidative Stress Levels

Oxidative stress levels were assessed using malondialdehyde (MDA) and total antioxidant capacity (T-AOC) kits (Abbkine, Santa Ana, CA, USA). Briefly, after drug administration, the supernatants were discarded. The volume required for 5 × 10^6^ cells was collected after counting, and the supernatant was centrifuged following ultrasonic disruption for 5 min. As instructed, reagents were added sequentially. The OD values were measured using a microplate reader.

### 2.12. Detection of ROS Level

Changes in ROS levels were assessed using the fluorescent probe DCFH-DA (Beyotime, China). DCFH-DA was diluted to a final concentration of 10 μmol/L using serum-free medium. Following removal of the cell culture medium, the appropriate amount of diluted DCFH-DA was added to the cells and incubated for 20 min. Fluorescence intensity was then observed using a fluorescence microscope.

### 2.13. Detection of Apoptosis

Following the instructions of the Annexin V-AbFluor™ 488/PI Dual Staining Apoptosis Kit (Abbkine, USA), cells were trypsinized to achieve a final volume containing 5 × 10^5^ cells. After centrifugation, the cells were resuspended in 1 × Annexin V Binding Buffer. Subsequently, 5 µL of Annexin V-AbFluor™ 488 and 2 µL of PI were added to the cells for 15 min. Finally, cells were analyzed by flow cytometry within 30 min.

### 2.14. Western Blotting

Total proteins were extracted using RIPA lysis buffer (Beyotime, China) with 1% protease inhibitor (Beyotime, China) and 1% phosphatase inhibitor (MedChemExpress, USA). Each protein sample was mixed with up-sampling buffer and denatured at 100 °C for 15 min. A total of 30 μg protein was loaded into each lane. Subsequently, protein samples were electrophoresed on an SDS-PAGE gel (8–12%), transferred to nitrocellulose membranes (Pall, Port Washington, NY, USA), and blocked with 5% BSA for 1 h. Subsequently, they were incubated with primary antibodies overnight at 4 °C. The antibodies used were as follows: CSNK1A1 (1:1000, ABclonal), Bax (1:8000, Wuhan, China, Proteintech), Bcl-2 (1:1500, Liyang, China, Affinity), caspase-3/p17/p19 (1:1500, Proteintech), AKT (1:1500, ABclonal), p-AKT (1:1500, ABclonal), PI3K (1:1500, ABclonal), p-PI3K (1:1000, Affinity), and GAPDH (1:100,000, Proteintech). Finally, the membranes were incubated with horseradish peroxidase-coupled goat anti-rabbit IgG for 1 h. Protein bands were visualized using an ECL kit (Beyotime, China), and analyzed with an automated gel image analysis system (Tanon, Shanghai, China).

### 2.15. Quantitative Real-Time Polymerase Chain Reaction (qRT-PCR) Analysis

Total RNA was extracted using the Trizol method. Subsequently, cDNA was synthesized using the cDNA Reverse Transcription Kit (Toyobo, Osaka, Japan). The synthesized cDNA was then subjected to quantitative qRT-PCR using the ChamQ Universal SYBR Green Master Mix (Vazyme, Nanjing, China). The cDNA content per well was 50 ng. The qRT-PCR was performed on LightCycler480 (Roche, Basel, Switzerland) to detect the cycling threshold (CT) value. GAPDH was utilized as an internal control, and the relative level of CSNK mRNA was calculated using 2^−ΔΔCT^ (Table 1).

### 2.16. Statistical Analyses

Statistical analysis was conducted using GraphPad Prism 8.0 (GraphPad Software, San Diego, CA, USA). Data are presented as mean ± standard deviation (SD). Student’s *t*-tests were employed to compare differences between two groups, while one-way analysis of variance (ANOVA) followed by Tukey’s multiple comparisons test was used for comparisons among multiple groups. *p* < 0.05 was considered statistically significant.

## 3. Results

### 3.1. ZEN Induces Mammary Gland Damage in Mice

**Figure 1:**
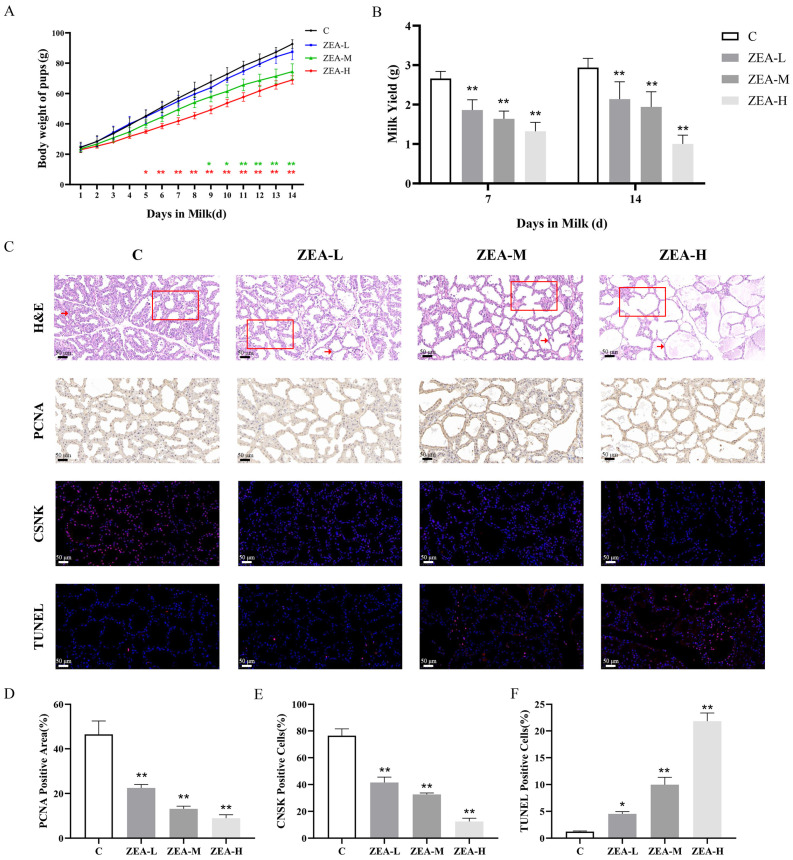
ZEA exposure affects lactation ability and induces mammary gland damage in lactating mice. (**A**) Daily body weight changes in different groups of young mice (*n* = 5). (**B**) Milk production in the mammary glands of female mice on days 7 and 14 of lactation (*n* = 5). (**C**) Mammary tissue hematoxylin and eosin (H&E) staining, PCNA immunohistochemistry, CSNK immunofluorescence, and fluorescent TUNEL staining (200×, scale bar = 50 μm), changes in follicular wall thickness are shown as arrows, and changes in follicular lumen are shown as squares. (**D**) Comparison of the PCNA positivity rate of mammary glands in each group by optical density. (**E**) CSNK immunofluorescence positive cell rate of mammary glands in each group. (**F**) Fluorescent TUNEL-positive cell rate of mammary glands in each group (*n* = 3; one-way ANOVA; * *p* < 0.05, ** *p* < 0.01 versus group C).

To determine whether ZEA damages mammary tissue in vivo, mice were administered 5 mg/kg, 10 mg/kg, and 20 mg/kg ZEA for 14 days. Firstly, we indirectly assessed milk production by measuring the body weights of pups on a daily basis (Figure 1A). The results showed that the body weights of the pups in the ZEA-H group were significantly lower on day 5 and highly significantly lower on day 6 of lactation, and the body weights of the pups in ZEA-M group were significantly lower on day 9 and highly significantly lower on day 10 of lactation. Milk production was highly significantly (Figure 1B) lower on day 7 and day 14 in the ZEA-L, ZEA-M, and ZEA-H groups. The exposure to ZEA in the ZEA-L group was significantly lower on day 7 and day 14 than that in the ZEA-H group (Figure 1B). This result suggests that ZEA exposure can significantly reduce lactation in the mammary gland.

Meanwhile, HE staining results (Figure 1C) showed that the mammary gland in group C was rich in follicular cells, and all ZEA-exposed groups showed a reduction in mammary epithelial cells, thinning of the follicular wall (shown by arrows), and enlargement of the follicular lumen (shown by squares). In addition, it was found that ZEA exposure can significantly reduce the expression of PCNA in mammary tissues, significantly reduce the CSNK protein positivity rate in mammary tissues, and elevate the TUNEL positivity rate in mammary tissues (Figure 1C–F). The above results indicated that different levels of ZEA exposure significantly induced mammary gland damage and reduced lactation capacity in lactating mice.

### 3.2. ZEA Inhibits MAC-T Cell Proliferation and Induces Apoptosis

**Figure 2:**
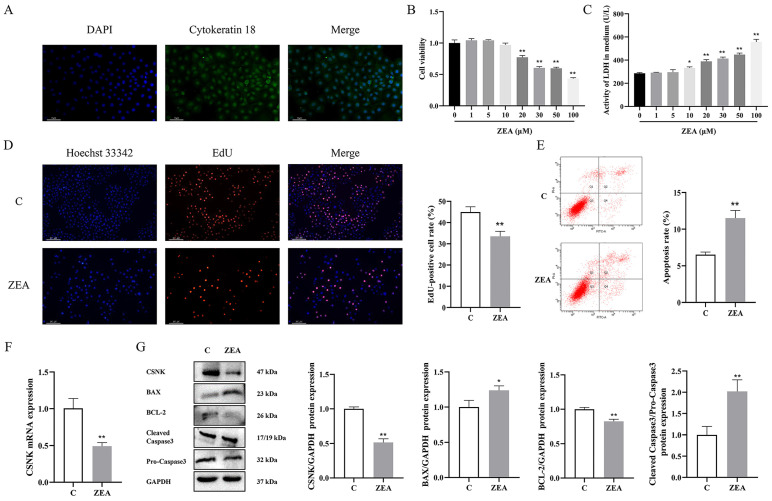
ZEA inhibits MAC-T cell proliferation and induces apoptosis. (**A**) MAC-T cell identification by immunofluorescence staining of cytokeratin 18 (200×, scale bar = 50 μm). (**B**) Changes in cell viability of MAC-T cells induced by ZEA for 24 h. (**C**) Changes in LDH viability in cell culture medium after 24 h of ZEA-induced MAC-T cells (*n* = 3; one-way ANOVA; * *p* < 0.05, ** *p* < 0.01 versus the group C). (**D**) The effect of ZEA on MAC-T cell proliferation was detected by EdU staining (200×, scale bar = 50 μm). (**E**) The effect of ZEA on the apoptosis level of MAC-T cells was detected by flow cytometry. (**F**) Effect of ZEA on CSNK mRNA levels in MAC-T cells. (**G**) Effect of ZEA on the protein levels of CSNK, BAX, BCL-2, and pro-/cleaved caspase-3 in MAC-T cells (*n* = 3; Student’s *t*-test; * *p* < 0.05, ** *p* < 0.01 versus the group C).

First, MAC-T cell identification was performed by immunofluorescence to detect the expression of cytokeratin 18 in cells (Figure 2A). We treated MAC-T with different concentrations of ZEA (0, 1, 5, 10, 20, 30, 50 and 100 μM) for 24 h. The results demonstrated significant changes in both cell viability and membrane integrity when exposed to 20 μM ZEA (Figure 2B,C). Consequently, 20 μM ZEA was selected for subsequent experiments. The effect of ZEA on MAC-T cell proliferation was evaluated using EdU. The results showed (Figure 2D) that the intensity of red fluorescence decreased significantly after ZEA exposure. The effect of ZEA on apoptosis of MAC-T cells was assessed using Annexin V-AbFluor™ 488/PI double staining. The results showed (Figure 2E) that ZEA-induced apoptosis was significantly elevated. Western blotting results indicated that ZEA significantly increased the expression of BAX and cleaved caspase-3, decreasing the expression of BCL-2 (Figure 2G). In addition, ZEA exposure caused significant reductions in levels of CSNK in MAC-T (Figure 1E). Collectively, the results suggest that exposure to ZEA inhibits the proliferation of MAC-T cells, induces apoptosis, and diminishes the production of CSNK.

### 3.3. ZEA Induces Oxidative Stress in MAC-T

**Figure 3:**
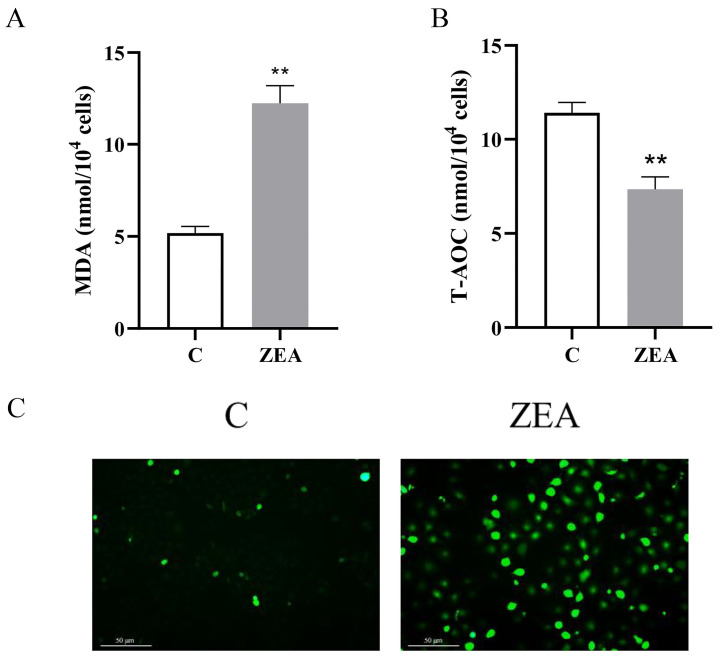
Effect of ZEA on oxidative stress levels in MAC-T cells. (**A**) Effect of ZEA on MDA levels in MAC-T cells. (**B**) Effect of ZEA on T-AOC levels in MAC-T cells (*n* = 3; Student’s *t*-test; ** *p* < 0.01 versus the group C). (**C**) Effect of ZEA on ROS levels in MAC-T cells (200×, scale bar = 50 μm).

In order to investigate how oxidative stress affects MAC-T proliferation and apoptosis, we examined the levels of oxidative stress in cells. The results (Figure 3A,B) demonstrated that ZEA significantly increased the MDA content and markedly inhibited the total antioxidant capacity. In addition, the average fluorescence intensity of ROS in MAC-T cells was significantly increased after ZEA treatment (Figure 3C). These findings suggest an elevation in cellular oxidative stress, a reduction in antioxidant capacity, and an accumulation of ROS within the cells upon exposure to ZEA.

### 3.4. ROS Accumulation Is Linked to ZEA-Induced Proliferation and Apoptosis of MAC-T

**Figure 4:**
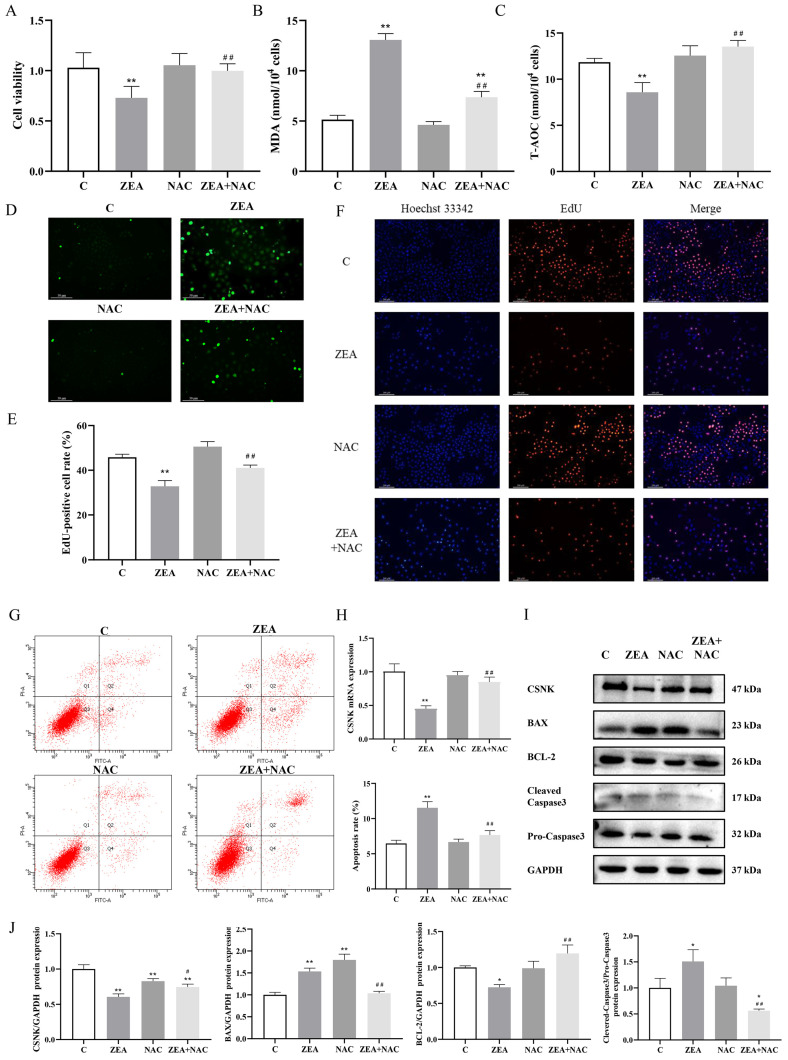
NAC reverses ZEA-regulated MAC-T cell proliferation and apoptosis. (**A**) Changes in MAC-T cell viability induced by ZEA with NAC. (**B**) Changes in MDA content in MAC-T cells after ZEA vs. NAC induction. (**C**) Changes in T-AOC content in MAC-T cells after ZEA vs. NAC induction. (**D**) Effect of ROS levels in MAC-T cells after ZEA vs. NAC induction (200×, scale bar = 50 μm). (**E**,**F**) Effect of ZEA with NAC-induced MAC-T cell proliferation detected by EdU staining (100×, scale bar = 200 μm). (**G**) The effect of ZEA on the level of apoptosis of MAC-T cells after NAC induction was detected by flow cytometry. (**H**) Effect of ZEA with CSNK mRNA levels in MAC-T cells after NAC induction. (**I**,**J**) Effects of CSNK, BAX, BCL-2, and pro-/cleaved caspase-3 expression levels in MAC-T cells after induction of ZEA with NAC (*n* = 3; one-way ANOVA; * *p* < 0.05, ** *p* < 0.01 versus group C; # *p* < 0.05, ## *p* < 0.01 versus the ZEA group).

Based on the preceding analysis, we hypothesized that ZEA-induced proliferation and apoptosis in MAC-T are mediated by ROS accumulation. To test this hypothesis, we employed NAC as a ROS scavenger. Our results demonstrated that pretreatment with 5 mM NAC for 2 h effectively reduced the accumulation of ROS (Figure 4D). Furthermore, the down-regulation of MAC-T cell viability induced by ZEA was significantly inhibited by NAC treatment (Figure 4A). Notably, the oxidative stress response of MAC-T was markedly reduced, and the antioxidant capacity was significantly elevated following NAC treatment (Figure 4B–D). Moreover, NAC treatment effectively reversed the alterations in MAC-T cell proliferation (Figure 4E,F) and apoptosis (Figure 4G) induced by ZEA exposure, while also up-regulating both mRNA and protein levels of CSNK (Figure 4H,I). Additionally, changes in apoptotic protein levels were assessed following NAC pretreatment (Figure 4I,J). As anticipated, pretreatment with NAC significantly attenuated the expression of BAX and cleaved caspase-3, and upregulated the expression of BCL-2. Based on the above results, NAC partially reversed the effects of ZEA on MAC-T viability, apoptosis, and proliferation.

### 3.5. ROS-Mediated PI3K/AKT Signaling Pathway Is Involved in ZEA Regulation of MAC-T

#### 3.5.1. ZEA Inhibits PI3K/AKT Signaling Pathway in MAC-T

**Figure 5:**
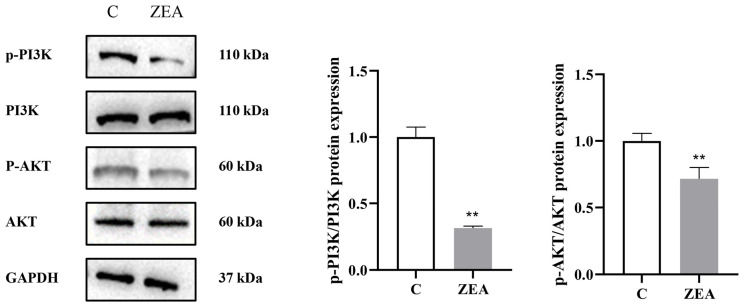
ZEA inhibits the PI3K/AKT signaling pathway in MAC-T. Effect of ZEA on the expression levels of p-PI3K/PI3K and p-AKT/AKT in MAC-T cells (*n* = 3; Student’s *t*-test; ** *p* < 0.01 versus the group C).

Accordingly, we investigated how ZEA affected the PI3K/AKT pathway in MAC-T. We found that ZEA treatment induced p-PI3K and p-AKT expression in MAC-T (Figure 5), suggesting that ZEA inhibited the pathway’s activation.

#### 3.5.2. Involvement of PI3K/AKT Signaling Pathway in the Regulation of MAC-T by ZEA

**Figure 6:**
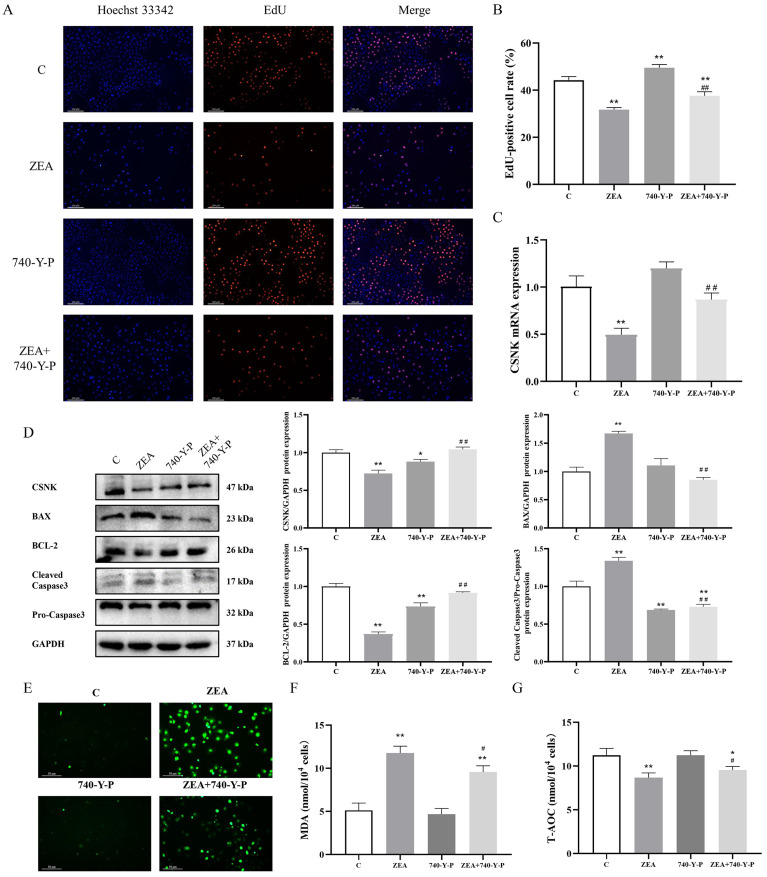
PI3K/AKT signaling pathway involved in ZEA regulation of MAC-T. (**A**,**B**) Effects of ZEA with 740-Y-P-induced proliferation levels in MAC-T cells (100×, scale bar = 200 μm). (**C**) Effects of ZEA on CSNK mRNA level in MAC-T cells after 740-Y-P induction. (**D**) Effects of CSNK, BAX, BCL-2, and pro-/cleaved caspase-3 expression levels in MAC-T cells after ZEA versus 740-Y-P induction. (**E**) Effect of ROS levels in MAC-T cells after ZEA vs. 740-Y-P induction (200×, scale bar = 50 μm). (**F**) Changes in MDA content in MAC-T cells after ZEA vs. 740-Y-P induction. (**G**) Changes in T-AOC content in MAC-T cells after ZEA vs. 740-Y-P induction. (*n* = 3; one-way ANOVA; * *p* < 0.05, ** *p* < 0.01 versus group C; # *p* < 0.05, ## *p* < 0.01 versus ZEA group).

To confirm that ZEA regulates MAC-T proliferation and apoptosis via the PI3K/AKT pathway, we utilized 740-Y-P as a PI3K activator. The activation effect of 740-Y-P on p-PI3K is shown in Supplementary Figure S1. EdU staining results demonstrated (Figure 6A,B) a significant elevation in the DNA replication ability with 740-Y-P treatment. Western blotting analysis (Figure 6D) revealed that treatment with 740-Y-P significantly reversed the ZEA-induced up-regulation of BAX and cleaved caspase-3, and mitigated the down-regulation of BCL-2. Moreover, 740-Y-P also attenuated the inhibitory effect on CSNK expression (Figure 6C,D). After pretreatment with 740-Y-P, the oxidative stress level of MAC-T cells was detected. The results showed that the MDA content of MAC-T cells in the ZEA + 740-Y-P group was significantly elevated compared with that of the C group and significantly reduced compared with that of the ZEA group; the T-AOC content was significantly reduced compared with that of the C group and significantly elevated compared with that of the ZEA group (Figure 6E–G). These findings suggest that the activation of PI3K effectively reverses the inhibitory effect of ZEA on proliferative ability, alleviates apoptosis, and upregulates the expression of CSNK.

#### 3.5.3. The Inhibitory Effect of ZEA on PI3K/AKT Signaling Pathway Is ROS-Dependent

**Figure 7:**
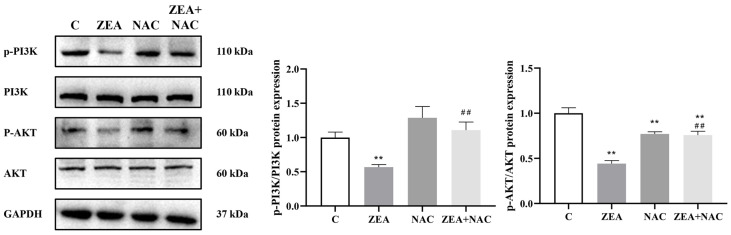
The inhibitory effect of ZEA on the PI3K/AKT signaling pathway is ROS-dependent. Effect of ZEA on the expression levels of p-PI3K/PI3K and p-AKT/AKT in MAC-T cells after NAC induction (*n* = 3; one-way ANOVA; ** *p* < 0.01 versus group C; ## *p* < 0.01 versus ZEA group).

In order to investigate whether ROS may act as an upstream regulator of ZEA-induced inhibition of the PI3K/AKT pathway, we treated MAC-T cells with NAC (Figure 7). The expression of p-PI3K and p-AKT was down-regulated after ZEA + NAC co-treatment compared to the single ZEA-treated group, indicating that NAC reversed the ZEA-induced inactivation of PI3K/AKT signaling pathway. Based on these findings, it appears that ZEA inhibits PI3K/AKT pathway activation in MAC-T cells by upregulating ROS levels.

## 4. Discussion

ZEA is a non-steroidal estrogenic mycotoxin commonly found in moldy feeds, capable of causing irreversible damage in both animals and humans. However, the precise mechanisms underlying ZEA-induced mammary gland damage remain unclear, and its effects on mammary gland function and milk production in lactating animals are still not well understood. In our study, lactating mice were exposed to varying doses of ZEA (5 mg/kg, 10 mg/kg, and 20 mg/kg), which induced decreased lactation performance, and thus slower body weight gain among their offspring. Additionally, ZEA disrupted the structure of mammary gland alveoli, decreased proliferation and CSNK expression of mammary epithelial cells, and promoted apoptosis in vivo. The in vitro experiments demonstrated that ZEA induced oxidative stress and elevated apoptosis levels in MAC-T cells, while inhibiting cell proliferation, the PI3K/AKT signaling pathway, and the expression of CSNK. Furthermore, the application of ROS scavengers and PI3K activators effectively reversed ZEA-induced damage in MAC-T cells. The results showed that ZEA induced mammary gland injury and inhibited the lactation function of mammary cells through the ROS-mediated PI3K/AKT signaling pathway.

Mycotoxins are significant contaminants in cereal feed supplies, with ZEA being one of the most notable. ZEA is an estrogen produced by fungi of genus *Fusarium*. Christiane Gruber-Dorninger et al. analyzed 74,821 samples of feed and feed ingredients from over 100 countries/regions worldwide between 2008 and 2017, finding that 45% of the samples were contaminated with ZEA, with the highest concentration reaching 105 mg/kg [4]. Similarly, Hao W et al. tested 9392 feed and feed ingredient samples from China from 2017 to 2021 and reported a contamination rate of 56.29%, with the maximum concentration reaching 11.245 mg/kg [40]. Additionally, Biscoto G L et al. evaluated 1749 cattle feed and feed ingredient samples, revealing that 62.5% were contaminated with ZEA, with the highest concentration recorded at 2503.86 μg/kg in the total mixed die [41]. These findings significantly exceed the maximum ZEA contamination levels established by European Union, which are 500 μg/kg for compound feeds and 3000 μg/kg for maize by-products. It has been shown that the feed intake of lactating mice increases by 4–5 times compared to their usual consumption [42]. According to Kun Pang et al., the feed intake of lactating KM mice ranges from approximately 10 to 22 g per day [43]. In the present study, after extensive literature review and conducting pre-experiments [34,44], we designed this experiment where lactating female mice were administered 5 mg/kg, 10 mg/kg, and 20 mg/kg of ZEA daily via force-feeding. In our study, different doses of ZEA significantly reduced lactation protein expression in mammary tissues, disrupted mammary alveolus structure, impaired milk production performance, and delayed offspring growth. Mammary gland development is driven by a balance between cell proliferation and apoptosis, and any disruption in this homeostasis can alter the cell population within the gland [45]. PCNA, a critical factor involved in DNA replication and repair, plays an essential role in promoting cell proliferation. ZEA exposure was found to reduce the expression of PCNA in mammary epithelial cells, impairing the gland’s proliferative capacity and inducing apoptosis in mammary tissues. Therefore, this study confirms that ZEA exposure impairs lactation ability in lactating mice.

Dairy cows are economically significant animals, and their milk production performance and milk quality are key indicators of their economic value. Fibroblasts, adipocytes, mammary epithelial cells, and myoepithelial cells compose mammary tissue. Mammary epithelial cells play an important role in both milk production and immunity during lactation, and their viability and number have a direct effect on milk protein synthesis [46]. CSNK is the most conserved casein gene and is indispensable during the lactation period [47]. The level of CSNK reflects the capacity of mammary epithelial cells to synthesize milk proteins [25]. Additionally, research has shown that excessive ROS levels result in increased intracellular oxidative stress, which disrupts proteins, DNA, and biomolecules, ultimately leading to apoptosis through the caspase pathway [48]. Notably, mammary epithelial cells are particularly sensitive to oxidative stress [49]. Elevated oxidative stress in mammary tissue can severely damage mammary epithelial cells, consequently reducing milk production [50]. MDA, a byproduct of lipid peroxidation, serves as an indicator of cellular damage, while the T-AOC reflects the overall antioxidant defense of the organism [51]. In our study, ZEA exposure significantly increased apoptosis, elevated ROS production, and raised MDA levels, while concurrently reducing CSNK content and T-AOC levels. These results suggest that ZEA induces oxidative damage, activates the caspase-dependent apoptotic pathway, promotes apoptosis, inhibits cell proliferation, and thereby impairs lactation function in MAC-T cells.

Due to the pivotal role oxidative stress plays in mammary gland injury in dairy cows, relieving oxidative stress could offer therapeutic benefits. NAC is a well-known antioxidant, effective in scavenging excess ROS across various tissues. For instance, in Parkinson’s disease, NAC was shown to inhibit neurodegenerative lesions by reducing ROS and restoring p38/Parkin-mediated mitochondrial function [52]. Similarly, Zhang et al. demonstrated that NAC effectively lowered ROS levels during the freezing and thawing of boar sperm, preserving sperm viability, maintaining plasma membrane integrity, and reducing apoptosis [53]. Xinyu Liu and colleagues found that NAC decreased oxidative stress and autophagy in ZEA-induced goat Sertoli cells by minimizing ROS production [29]. In the present study, to further explore the role of ROS in ZEA-regulated proliferation and apoptosis of MAC-T cells, we employed NAC to scavenge ROS. Our findings revealed that NAC treatment reduced MDA levels and enhanced T-AOC levels, effectively mitigating cellular oxidative stress and boosting antioxidant capacity. Moreover, NAC ameliorated ZEA-induced declines in MAC-T cell viability, while inhibiting apoptosis and restoring normal cell proliferation and CSNK levels. These results indicate that ZEA’s apoptosis-promoting and proliferation-inhibiting effects on MAC-T cells are primarily mediated by excessive ROS, and NAC counters ZEA-induced cytotoxicity.

The PI3K/AKT signaling pathway plays a crucial role in regulating key cellular processes such as proliferation, cell cycle control, apoptosis, and protein synthesis [22]. It has been observed that ROS accumulation, induced by factors such as high glucose, inflammatory agents, and toxicants, can disrupt the normal functioning of the PI3K/AKT pathway. For example, high glucose-induced downregulation of SIRT1 inhibits PI3K/AKT, leading to inflammation, apoptosis, and delayed wound healing [54]. Additionally, cadmium exposure causes its accumulation in the porcine thymus, promoting apoptosis through the ROS-dependent PTEN/PI3K/AKT pathway [55]. In this study, we found that ZEA significantly inhibited the expression of p-PI3K and p-AKT in MAC-T cells. The use of 740-Y-P, a potent activator of PI3K phosphorylation, effectively mitigated ZEA’s inhibitory effects on MAC-T cell proliferation and CSNK expression. Simultaneously, it significantly suppressed apoptosis mediated by the cysteine asparaginase pathway. In addition, pretreatment with 740-Y-P was only able to partially alleviate the level of oxidative stress induced by ZEA, and the antioxidant level of the cells was significantly lower than that in group C. However, inhibition of ROS production through NAC reactivated the PI3K/AKT pathway, thereby reducing ZEA-induced cellular damage. Thus, we conclude that ZEA induces apoptosis and suppresses cell proliferation by modulating the PI3K/AKT signaling pathway through ROS regulation.

## 5. Conclusions

In conclusion, our findings demonstrate that ZEA exposure leads to significant mammary gland damage and impairs lactation function. ZEA induces oxidative stress and apoptosis in mammary epithelial cells while inhibiting cell proliferation. Importantly, our study revealed that ZEA’s damaging effects on mammary epithelial cells are mediated via the ROS-PI3K/AKT pathway. By scavenging ROS and activating PI3K, the cytotoxic effects of ZEA can be effectively mitigated, highlighting that PI3K/AKT may be a potential target for developing therapeutic interventions to prevent and manage ZEA-induced mammary gland injury in dairy cows. These results contribute to a deeper understanding of the molecular mechanisms underlying ZEA-induced mammary gland injury and provide a theoretical foundation for creating safer feeds and protective agents against ZEA toxicity. However, further studies using in vivo or ex vivo methods in dairy cows are needed to determine whether this pathway can reduce ZEA residues in milk and develop more comprehensive protective measures.

## Figures and Tables

**Table 1:** Primer sequence.

Target Gene	Forward Primer	Reverse Primer
*CSNK* (NC_037333.1)	CCAGGAGCAAAACCAAGAAC	TGCAACTGGTTTCTGTTGGT
*GAPDH* (NC_037332.1)	ACTGGCGTCTTCACCACCAT	AAGGCCATGCCAGTGAGCTT

## Data Availability

The data used to support the findings of this study are available from the corresponding author upon request.

## Supplementary Materials

The following supporting information can be downloaded at: www.mdpi.com/article/10.3390/ani15071050/s1, Figure S1: Examination of the inhibitory effect of 740-Y-P on the PI3K/AKT pathway.

## Abbreviations

AKT, protein kinase B; CSNK, κ-casein; LDH, lactate dehydrogenase; MAC-T, mammary epithelial cells; MDA, malondialdehyde; NAC, N-acetylcysteine; OD, optical density; PCNA, proliferating cell nuclear antigen; PI3K, phosphatidylinositol 3-kinase; ROS, reactive oxygen species; T-AOC, total antioxidant capacity; ZEA, zearalenone.

## References

[1] Latham R.L., Boyle J.T., Barbano A., Loveman W.G., Brown N.A. (2023). Diverse mycotoxin threats to safe food and feed cereals. Essays Biochem..

[2] Marins-Goncalves L., Martins Ferreira M., Rocha Guidi L., De Souza D. (2023). Is chemical analysis suitable for detecting mycotoxins in agricultural commodities and foodstuffs?. Talanta.

[3] Ryu D., Jackson L.S., Bullerman L.B. (2002). Effects of processing on zearalenone. Adv. Exp. Med. Biol..

[4] Gruber-Dorninger C., Jenkins T., Schatzmayr G. (2019). Global Mycotoxin Occurrence in Feed: A Ten-Year Survey. Toxins.

[5] Rai A., Das M., Tripathi A. (2020). Occurrence and toxicity of a fusarium mycotoxin, zearalenone. Crit. Rev. Food Sci. Nutr..

[6] Seeling K., Danicke S., Ueberschar K.H., Lebzien P., Flachowsky G. (2005). On the effects of Fusarium toxin-contaminated wheat and the feed intake level on the metabolism and carry over of zearalenone in dairy cows. Food Addit. Contam..

[7] Omar S.S. (2021). Prevalence, level and health risk assessment of mycotoxins in the fried poultry eggs from Jordan. Environ. Res..

[8] Liu J., Applegate T. (2020). Zearalenone (ZEN) in Livestock and Poultry: Dose, Toxicokinetics, Toxicity and Estrogenicity. Toxins.

[9] Rogowska A., Pomastowski P., Sagandykova G., Buszewski B. (2019). Zearalenone and its metabolites: Effect on human health, metabolism and neutralisation methods. Toxicon.

[10] Mauro T., Hao L., Pop L.C., Buckley B., Schneider S.H., Bandera E.V., Shapses S.A. (2018). Circulating zearalenone and its metabolites differ in women due to body mass index and food intake. Food Chem. Toxicol..

[11] Rubert J., Leon N., Saez C., Martins C.P., Godula M., Yusa V., Manes J., Soriano J.M., Soler C. (2014). Evaluation of mycotoxins and their metabolites in human breast milk using liquid chromatography coupled to high resolution mass spectrometry. Anal. Chim. Acta.

[12] Alshannaq A., Yu J.H. (2017). Occurrence, Toxicity, and Analysis of Major Mycotoxins in Food. Int. J. Environ. Res. Public. Health.

[13] Fleck S.C., Churchwell M.I., Doerge D.R., Teeguarden J.G. (2016). Urine and serum biomonitoring of exposure to environmental estrogens II: Soy isoflavones and zearalenone in pregnant women. Food Chem. Toxicol..

[14] Falkauskas R., Bakutis B., Jovaisiene J., Vaiciulience G., Gerulis G., Kerziene S., Jaceviciene I., Jacevicius E., Baliukoniene V. (2022). Zearalenone and Its Metabolites in Blood Serum, Urine, and Milk of Dairy Cows. Animals.

[15] Goyarts T., Danicke S., Brussow K.P., Valenta H., Ueberschar K.H., Tiemann U. (2007). On the transfer of the Fusarium toxins deoxynivalenol (DON) and zearalenone (ZON) from sows to their fetuses during days 35–70 of gestation. Toxicol. Lett..

[16] Mahato D.K., Devi S., Pandhi S., Sharma B., Maurya K.K., Mishra S., Dhawan K., Selvakumar R., Kamle M., Mishra A.K. (2021). Occurrence, Impact on Agriculture, Human Health, and Management Strategies of Zearalenone in Food and Feed: A Review. Toxins.

[17] Wang S., Fu W., Zhao X., Chang X., Liu H., Zhou L., Li J., Cheng R., Wu X., Li X. (2022). Zearalenone disturbs the reproductive-immune axis in pigs: The role of gut microbial metabolites. Microbiome.

[18] Malekinejad H., Schoevers E.J., Daemen I.J., Zijlstra C., Colenbrander B., Fink-Gremmels J., Roelen B.A. (2007). Exposure of oocytes to the Fusarium toxins zearalenone and deoxynivalenol causes aneuploidy and abnormal embryo development in pigs. Biol. Reprod..

[19] Lo E.K.K., Wang X., Lee P.K., Wong H.C., Lee J.C., Gomez-Gallego C., Zhao D., El-Nezami H., Li J. (2023). Mechanistic insights into zearalenone-accelerated colorectal cancer in mice using integrative multi-omics approaches. Comput. Struct. Biotechnol. J..

[20] Ma Z., Li Q., Xu H., Li Y., Wang S., Xiong Y., Lan D., Li j., Xiong X., Fu W. (2024). Zearalenone triggers programmed cell death and impairs milk fat synthesis via the AKT-mTOR-PPARgamma-ACSL4 pathway in bovine mammary epithelial cells. J. Anim. Sci..

[21] Ersahin T., Tuncbag N., Cetin-Atalay R. (2015). The PI3K/AKT/mTOR interactive pathway. Mol. Biosyst..

[22] Ling M., Quan L., Lai X., Lang L., Li F., Yang X., Fu Y., Feng S., Yi X., Zhu C. (2021). VEGFB Promotes Myoblasts Proliferation and Differentiation through VEGFR1-PI3K/Akt Signaling Pathway. Int. J. Mol. Sci..

[23] Xu Z.J., Liu M., Niu Q.J., Huang Y.X., Zhao L., Lei X.G., Sun L.H. (2023). Both selenium deficiency and excess impair male reproductive system via inducing oxidative stress-activated PI3K/AKT-mediated apoptosis and cell proliferation signaling in testis of mice. Free Radic. Biol. Med..

[24] Lin X., Wang W., Chang X., Chen C., Guo Z., Yu G., Shao W., Wu S., Zhang Q., Zheng F. (2024). ROS/mtROS promotes TNTs formation via the PI3K/AKT/mTOR pathway to protect against mitochondrial damages in glial cells induced by engineered nanomaterials. Part. Fibre Toxicol..

[25] Li H., Liu X., Wang Z., Lin X., Yan Z., Cao Q., Zhao M., Shi K. (2017). MEN1/Menin regulates milk protein synthesis through mTOR signaling in mammary epithelial cells. Sci. Rep..

[26] Parveen S., Zhu P., Shafique L., Lan H., Xu D., Ashraf S., Ashraf S., Sherazi M., Liu Q. (2023). Molecular Characterization and Phylogenetic Analysis of Casein Gene Family in Camelus ferus. Genes.

[27] Staiger E.A., Thonney M.L., Buchanan J.W., Rogers E.R., Oltenacu P.A., Mateescu R.G. (2010). Effect of prolactin, beta-lactoglobulin, and kappa-casein genotype on milk yield in East Friesian sheep. J. Dairy. Sci..

[28] Pizarro M.G., Landi V., Navas F.J., Leon J.M., Martinez A., Fernandez J., Delgado J.V. (2020). Nonparametric analysis of casein complex genes’ epistasis and their effects on phenotypic expression of milk yield and composition in Murciano-Granadina goats. J. Dairy. Sci..

[29] Liu X., Xi H., Han S., Zhang H., Hu J. (2023). Zearalenone induces oxidative stress and autophagy in goat Sertoli cells. Ecotoxicol. Environ. Saf..

[30] Abuelo A., Hernandez J., Benedito J.L., Castillo C. (2015). The importance of the oxidative status of dairy cattle in the periparturient period: Revisiting antioxidant supplementation. J. Anim. Physiol. Anim. Nutr..

[31] Bai J., Zhou Y., Luo X., Hai J., Si X., Li J., Fu H., Dai Z., Yang Y., Wu Z. (2022). Roles of stress response-related signaling and its contribution to the toxicity of zearalenone in mammals. Compr. Rev. Food Sci. Food Saf..

[32] Feng Y.Q., Zhao A.H., Wang J.J., Tian Y., Yan Z.H., Dri M., Shen W., De Felici M., Li L. (2022). Oxidative stress as a plausible mechanism for zearalenone to induce genome toxicity. Gene.

[33] Guo Z., Gao S., Ouyang J., Ma L., Bu D. (2021). Impacts of Heat Stress-Induced Oxidative Stress on the Milk Protein Biosynthesis of Dairy Cows. Animals.

[34] Yang C., Chen Y., Yang M., Li J., Wu Y., Fan H., Kong X., Ning C., Wang S., Xiao W. (2023). Betulinic acid alleviates zearalenone-induced uterine injury in mice. Environ. Pollut..

[35] Gao X., Sun L., Zhang N., Li C., Zhang J., Xiao Z., Qi D. (2017). Gestational Zearalenone Exposure Causes Reproductive and Developmental Toxicity in Pregnant Rats and Female Offspring. Toxins.

[36] Lin J., Zuo C., Liang T., Huang Y., Kang P., Xiao K., Liu Y. (2022). Lycopene alleviates multiple-mycotoxin-induced toxicity by inhibiting mitochondrial damage and ferroptosis in the mouse jejunum. Food Funct..

[37] Gao X., Xiao Z., Li C., Zhang J., Zhu L., Sun L., Zhang N., Khalil M.M., Rajput S.A., Qi D. (2018). Prenatal exposure to zearalenone disrupts reproductive potential and development via hormone-related genes in male rats. Food Chem. Toxicol..

[38] Wu K., Jia S., Xue D., Rajput S.A., Liu M., Qi D., Wang S. (2022). Dual effects of zearalenone on aflatoxin B1-induced liver and mammary gland toxicity in pregnant and lactating rats. Ecotoxicol. Environ. Saf..

[39] Yan X.R., Shi T., Xiao J.Y., Liu Y.F., Zheng H.L. (2022). In Vitro transdifferentiated signatures of goat preadipocytes into mammary epithelial cells revealed by DNA methylation and transcriptome profiling. J. Biol. Chem..

[40] Hao W., Guan S., Li A., Wang J., An G., Hofstetter U., Schatzmayr G. (2023). Mycotoxin Occurrence in Feeds and Raw Materials in China: A Five-Year Investigation. Toxins.

[41] Biscoto G.L., Salvato L.A., Alvarenga E.R., Dias R.R.S., Pinheiro G.R.G., Rodrigues M.P., Pinto P.N., Freitas R.P., Keller K.M. (2022). Mycotoxins in Cattle Feed and Feed Ingredients in Brazil: A Five-Year Survey. Toxins.

[42] Zhao Z.J., Derous D., Gerrard A., Wen J., Liu X., Tan S., Hambly C., Speakman J.R. (2020). Limits to sustained energy intake. XXX. Constraint or restraint? Manipulations of food supply show peak food intake in lactation is constrained. J. Exp. Biol..

[43] Pang K., Zhu Z., Zhu S., Han L. (2019). A high dose of conjugated linoleic acid increases fatty liver and insulin resistance in lactating mice. PLoS ONE.

[44] Wu J., Li J., Liu Y., Liao X., Wu D., Chen Y., Liang Z., Yuan Z., Li R., Yi J. (2021). Tannic acid repair of zearalenone-induced damage by regulating the death receptor and mitochondrial apoptosis signaling pathway in mice. Environ. Pollut..

[45] Li Y., Cao Y., Wang J., Fu S., Cheng J., Ma L., Zhang Q., Guo W., Kan X., Liu J. (2020). Kp-10 promotes bovine mammary epithelial cell proliferation by activating GPR54 and its downstream signaling pathways. J. Cell Physiol..

[46] Lee H., An G., Lim W., Song G. (2022). Pendimethalin exposure induces bovine mammary epithelial cell death through excessive ROS production and alterations in the PI3K and MAPK signaling pathways. Pestic. Biochem. Physiol..

[47] Shekar P.C., Goel S., Rani S.D., Sarathi D.P., Alex J.L., Singh S., Kumar S. (2006). kappa-casein-deficient mice fail to lactate. Proc. Natl. Acad. Sci. USA.

[48] Wang B., Wang Y., Zhang J., Hu C., Jiang J., Li Y., Peng Z. (2023). ROS-induced lipid peroxidation modulates cell death outcome: Mechanisms behind apoptosis, autophagy, and ferroptosis. Arch. Toxicol..

[49] Liu L., Lin Y., Liu L., Bian Y., Zhang L., Gao X., Li Q. (2015). 14-3-3gamma Regulates Lipopolysaccharide-Induced Inflammatory Responses and Lactation in Dairy Cow Mammary Epithelial Cells by Inhibiting NF-kappaB and MAPKs and Up-Regulating mTOR Signaling. Int. J. Mol. Sci..

[50] Gannuscio R., Ponte M., Di Grigoli A., Maniaci G., Di Trana A., Bacchi M., Alabiso M., Bonanno A., Todaro M. (2022). Feeding Dairy Ewes with Fresh or Dehydrated Sulla (*Sulla coronarium* L.) Forage. 1. Effects on Feed Utilization, Milk Production, and Oxidative Status. Animals.

[51] Wang H., Hao W., Yang L., Li T., Zhao C., Yan P., Wei S. (2022). Procyanidin B2 Alleviates Heat-Induced Oxidative Stress through the Nrf2 Pathway in Bovine Mammary Epithelial Cells. Int. J. Mol. Sci..

[52] Chen J., Li M., Zhou X., Xie A., Cai Z., Fu C., Peng Y., Zhang H., Liu L. (2021). Rotenone-Induced Neurodegeneration Is Enabled by a p38-Parkin-ROS Signaling Feedback Loop. J. Agric. Food Chem..

[53] Zhang B., Wang Y., Wu C., Qiu S., Chen X., Cai B., Xie H. (2021). Freeze-thawing impairs the motility, plasma membrane integrity and mitochondria function of boar spermatozoa through generating excessive ROS. BMC Vet. Res..

[54] Chen K., Li Y., Zhang X., Ullah R., Tong J., Shen Y. (2022). The role of the PI3K/AKT signalling pathway in the corneal epithelium: Recent updates. Cell Death Dis..

[55] Zhang Y., Liu Z., Lan J., Wang J., Shen Z., Shi G., Li S. (2021). Cadmium induces the thymus apoptosis of pigs through ROS-dependent PTEN/PI3K/AKT signaling pathway. Environ. Sci. Pollut. Res. Int..

