# Supplementary figures and images for: Zearalenone Depresses Lactation Capacity Through the ROS-Mediated PI3K/AKT Pathway
Source: Animals (Basel). 2025 Apr 4;15(7):1050. doi: 10.3390/ani15071050 (PMC11987832; doi:10.3390/ani15071050)

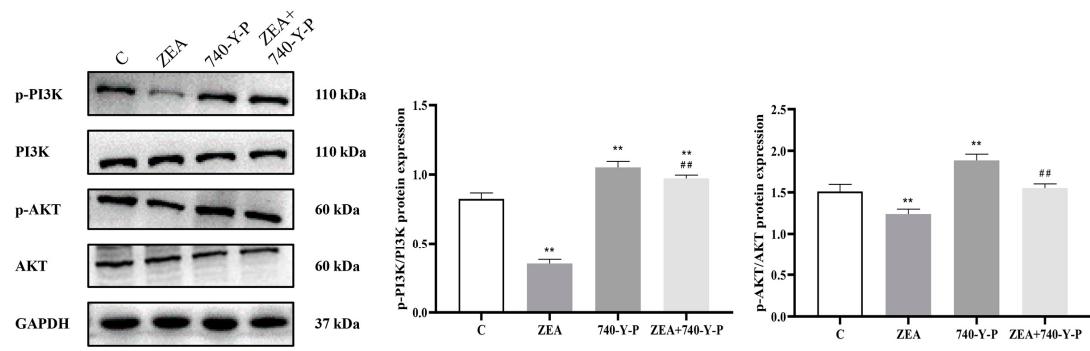

Figure S1: Examination of the inhibitory effect of 740-Y-P on the PI3K/AKT pathway.

Supplement: Supplementary file 1 [file animals-15-01050-s001.zip › Supplementary Figure S1.pdf]
